# Extraordinary Optical Transmission by Hybrid Phonon–Plasmon Polaritons Using hBN Embedded in Plasmonic Nanoslits

**DOI:** 10.3390/nano11061567

**Published:** 2021-06-14

**Authors:** Shinpei Ogawa, Shoichiro Fukushima, Masaaki Shimatani

**Affiliations:** Mitsubishi Electric Corporation, Advanced Technology R&D Center, 8-1-1 Tsukaguchi-Honmachi, Amagasaki 661-8661, Japan; Fukushima.Shoichiro@cb.MitsubishiElectric.co.jp (S.F.); Shimatani.Masaaki@bk.MitsubishiElectric.co.jp (M.S.)

**Keywords:** extraordinary optical transmission, hexagonal boron nitride, hybridization, hyperbolic phonon polaritons, nanoslits, surface plasmon resonances

## Abstract

Hexagonal boron nitride (hBN) exhibits natural hyperbolic dispersion in the infrared (IR) wavelength spectrum. In particular, the hybridization of its hyperbolic phonon polaritons (HPPs) and surface plasmon resonances (SPRs) induced by metallic nanostructures is expected to serve as a new platform for novel light manipulation. In this study, the transmission properties of embedded hBN in metallic one-dimensional (1D) nanoslits were theoretically investigated using a rigorous coupled wave analysis method. Extraordinary optical transmission (EOT) was observed in the type-II Reststrahlen band, which was attributed to the hybridization of HPPs in hBN and SPRs in 1D nanoslits. The calculated electric field distributions indicated that the unique Fabry–Pérot-like resonance was induced by the hybridization of HPPs and SPRs in an embedded hBN cavity. The trajectory of the confined light was a zigzag owing to the hyperbolicity of hBN, and its resonance number depended primarily on the aspect ratio of the 1D nanoslit. Such an EOT is also independent of the slit width and incident angle of light. These findings can not only assist in the development of improved strategies for the extreme confinement of IR light but may also be applied to ultrathin optical filters, advanced photodetectors, and optical devices.

## 1. Introduction

Polaritons in two-dimensional (2D) materials are very important in the field of nanophotonics in terms of both pure scientific interest and novel applications [1]. Alongside surface plasmon polaritons (SPPs) in graphene [2,3,4], hyperbolic phonon polaritons (HPPs) in hexagonal boron nitride (hBN) have drawn significant interest [5]. hBN is a van der Waals material that is expected to not only be a highly suitable insulator and encapsulation layer for graphene [6,7,8] but also a natural hyperbolic material [5,9]. hBN exhibits a natural hyperbolic dispersion relation in two infrared (IR) wavelength regions [9,10,11], although the in-plane and out-of-plane permittivity in each region is significantly different and opposite in terms of their sign [10]. This unique hyperbolic dispersion relation can enhance light–matter interactions, such as the extreme confinement of IR light [12,13] and hybridization of SPPs and HPPs [14,15,16,17].

Although artificially engineered hyperbolic metamaterials have typically been considered as the only solution for realizing hyperbolic dispersion relations [18], complicated structures such as subwavelength multilayer structures [19,20] or multilayer fishnets [21] are required. In contrast, hBN exhibits two distinct Reststrahlen (RS) bands, formed owing to the anisotropy of hBN, where natural hyperbolic dispersion occurs in the IR wavelength spectrum. hBN has been applied effectively in various applications, including perfect absorbers [22,23,24,25,26], thermal emitters [27,28], sub-diffraction imaging and focusing [29,30], waveguides [31,32,33], single photon emitters [34,35], molecular sensing using metasurfaces [36], and optical cavities using photonic crystals [37]. In particular, the hybridization of surface plasmon resonances (SPRs) induced by metallic nanostructures and HPPs in hBN serves as a new platform for novel light manipulation. Metallic nanostructures, such as 2D nanoholes and one-dimensional (1D) gratings, are well-known structures for implementing high-efficiency light control using SPPs, such as in extraordinary optical transmission (EOT) [38,39,40] and wavelength-selective perfect absorption/emission [41,42,43]. In addition, spectral filters using SPPs are highly promising for a wide range of applications, such as spectral imaging [44,45,46,47] and complementary metal oxide semiconductor sensors [48,49].

However, the effect of hBN in plasmonic structures, specifically in EOT, has not been fully investigated. EOT in the IR wavelength region is very important for realizing various optical filter applications. Therefore, in this study, hBN embedded in 1D nanoslits with a high aspect ratio (narrow slit width and high slit height) is proposed. Such 1D nanoslits produce EOT due to the hybridization of the Fabry–Pérot resonances of SPRs [40]. By studying the effect of hBN on EOT, the hybridization of HPPs in hBN, and the Fabry–Pérot resonances of SPRs, the coupling between the HPPs in hBN and the SPRs in 1D nanoslits is numerically investigated. The remainder of this paper is arranged as follows: Section 2 presents the materials and calculation model used, Section 3 compares the transmittances of the 1D nanoslits using Si as the isotropic material and hBN as the anisotropic dielectric, and Section 4 concludes the paper.

## 2. Material and Calculation Model

**Figure 1:**
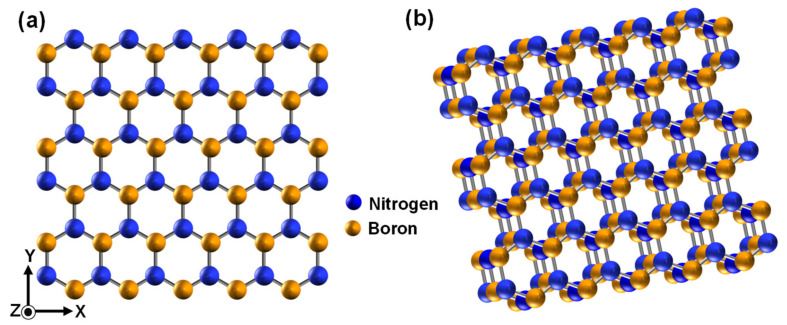
Schematic of (**a**) monolayer and (**b**) multilayer hBN.

Figure 1a,b illustrate the schematic of monolayer and multilayer hBNs, respectively. In this study, in-plane and out-of-plane were defined as parallel and normal to the x-y plane, respectively.

There are two types of phonon modes for hBN in IR wavelengths: one is an out-of-plane (∥) mode with ω_TO_ of 780 cm^−1^ and ω_LO_ of 830 cm^−1^; the other is an in-plane (⊥) mode with ω_TO_ of 1370 cm^−1^ and ω_LO_ of 1610 cm^−1^ [14]. Therefore, hBN has two anisotropic permittivities, $\epsilon_{∥}$ and $\epsilon_{⊥}$. From reference [14], the anisotropic permittivity of hBN is given by

(1) $$\epsilon_{m}=\epsilon_{\infty ,\;m}+\epsilon_{\infty ,\;m}\times \frac{{\left(\omega_{LO,\;m}\right)}^{2}-{\left(\omega_{TO,\;m}\right)}^{2}}{{\left(\omega_{TO,\;m}\right)}^{2}-\omega^{2}-i\omega \Gamma_{m}},$$

where m = ∥, ⊥. $\epsilon_{\infty ,\;⊥}=4.87,\;\epsilon_{\infty ,\;∥}=2.95,\;\Gamma_{⊥}=5\;cm^{-1},\;\mathrm{and}\;\Gamma_{∥}=4\;cm^{-1}$. These values were taken from reference [10].

**Figure 2:**
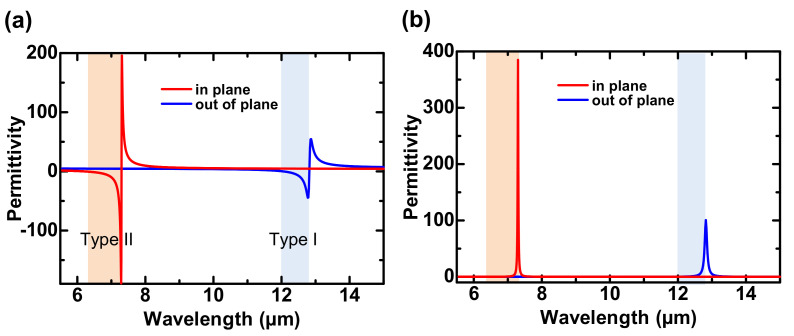
(**a**) Real and (**b**) imaginary parts of permittivity of hBN and two RS bands.

Figure 2a,b show the calculated real and imaginary parts of $\epsilon_{∥}$ and $\epsilon_{⊥}$, respectively. As seen in Figure 2a,b, two distinct RS bands formed owing to the anisotropy of hBN. The longer and shorter wavelength RS bands correspond to type-I $\left(\epsilon_{∥}\;<0,\epsilon_{⊥}\;>0\right)$ and type-II $\left(\epsilon_{⊥}\;<0,\epsilon_{∥}\;>0\right)$, respectively.

**Figure 3:**
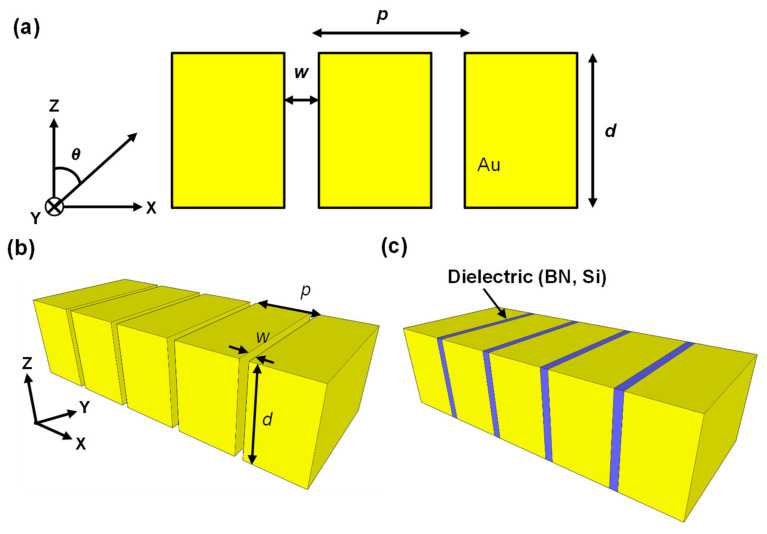
(**a**) Cross-sectional view of 1D nanoslits with a high aspect ratio, (**b**) 1D nanoslits without an embedded dielectric, and (**c**) 1D nanoslits with an embedded dielectric.

Figure 3a depicts a cross-sectional image of 1D nanoslits with a high aspect ratio, while Figure 3b,c depict a schematic of 1D nanoslits without and with an embedded dielectric, respectively. In this study, the length of the nanoslits in the y-direction was assumed to be infinite. The 1D nanoslits were based on Au; we used Au because it is the most conventional plasmonic nanoslit material [41,42,43,44]. Highly doped semiconductors such as ZnO, GaAs, and indium tin oxide can also be used as plasmonic material. The period, depth, and width of the nanoslits and the angle of the incident light were defined as *p*, *d*, *w*, and *θ*, respectively. The permittivity of Au was taken from reference [50]. Si and hBN were used as the isotropic and anisotropic dielectrics, respectively. *p* was fixed at 1.25 μm in this study.

A rigorous coupled wave analysis method [51] was used for all numerical calculations in this study. Additionally, the transverse magnetic (TM) mode, where the electric field is parallel to the x-axis, was used in this study because only the TM mode excites SPPs in 1D periodic structures [43]. Thus, the type-II RS band was primarily investigated.

## 3. Results and Discussion

### 3.1. Isotropic Materials

**Figure 4:**
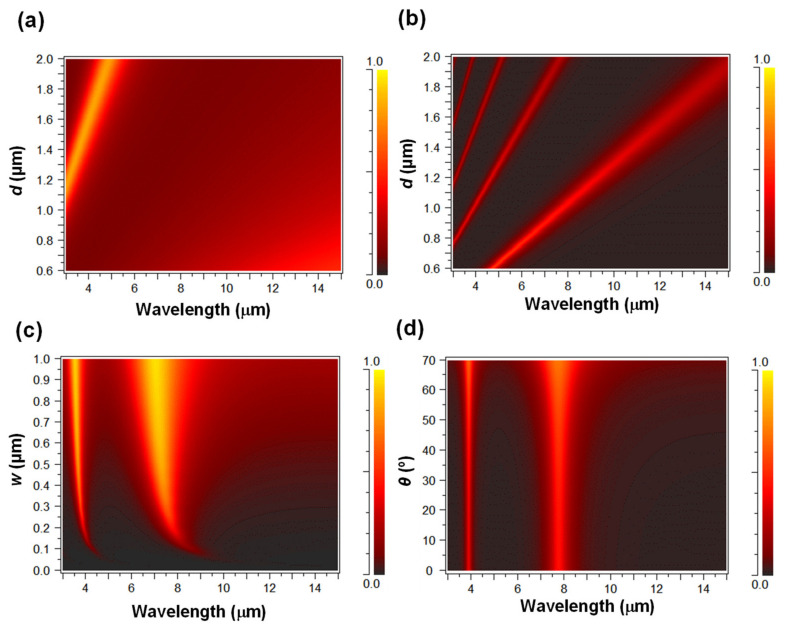
Calculated transmittance as a function of wavelength and (**a**) d for 1D nanoslits with air, (**b**) d for 1D nanoslits with Si, (**c**) *w* for 1D nanoslits with Si, and (**d**) *θ* for 1D nanoslits with Si. The color scale represents the transmittance.

First, the transmittance of 1D nanoslits without a dielectric and with Si as an isotropic material was investigated as a reference for the study of the anisotropic hBN. *w* and *θ* were fixed at 200 nm and 0°, respectively. Figure 4a,b show the calculated transmittance as a function of wavelength and *d* for 1D nanoslits with an air and with Si, respectively. Figure 4c,d show the calculated transmittance as a function of wavelength and *w* with a fixed *d* and *θ* of 1.0 μm and 0°, respectively, and *θ* with a fixed *d* and *w* of 1.0 μm and 200 nm for 1D nanoslits with Si, respectively.

Figure 4a,b clearly show that EOT was observed in both structures, and the enhanced transmittance was mainly proportional to *d*. Figure 4c,d show that the wavelength of EOT is nearly independent of *w* and *θ*. These properties are attributed to the Fabry–Pérot resonances of SPRs formed inside the slits [40].

### 3.2. Anisotropic hBN

**Figure 5:**
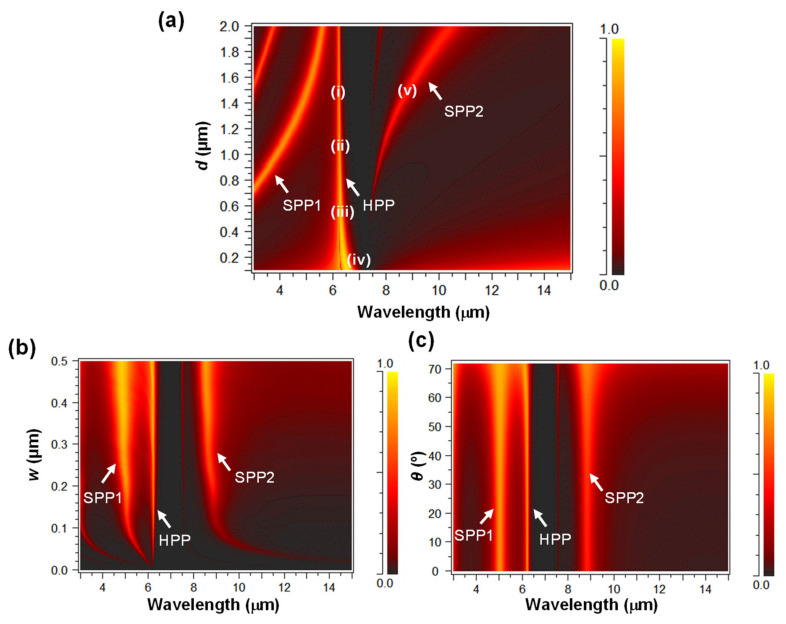
Calculated transmittance as a function of wavelength and (**a**) *d*, (**b**) *w*, and (**c**) *θ* for 1D nanoslits with hBN. The color scale represents the transmittance.

One-dimensional nanoslits with embedded hBN were investigated. The stacking direction of hBN is defined as parallel to the z-axis. Figure 5a–c show the calculated transmittance as a function of wavelength and *d* with a fixed *w* and *θ* of 200 nm and 0°, *w* with fixed *d* and *θ* of 1.5 μm and 0°, and *θ* with fixed *d* and w of 1.5 μm and 200 nm, respectively.

Figure 5a clearly indicates that the type-II RS band of hBN strongly couples SPP modes and induces Rabi splitting at a wavelength of approximately 6.5 μm. As a result, EOT of HPP is produced, and the SPP mode is split into SPP1 and SPP2, as denoted in Figure 5a. The HPP modes are nearly wavelength independent and are inside the type-II RS band of hBN. Figure 5b,c also shows that SPP1, SPP2, and HPP are independent of *w* and *θ*, where the aspect ratio *d*/*w* is sufficiently large with a small *w* because the effect of *d* is dominant for SPRs in nanoslits [43]. The SPP1 and SPP2 modes are nearly proportional to *d* and are outside the type-II RS band of hBN. Therefore, both the SPP1 and SPP2 modes are attributed to the Fabry–Pérot resonances of SPPs induced in 1D nanoslits, as discussed in Section 3.1.

**Figure 6:**
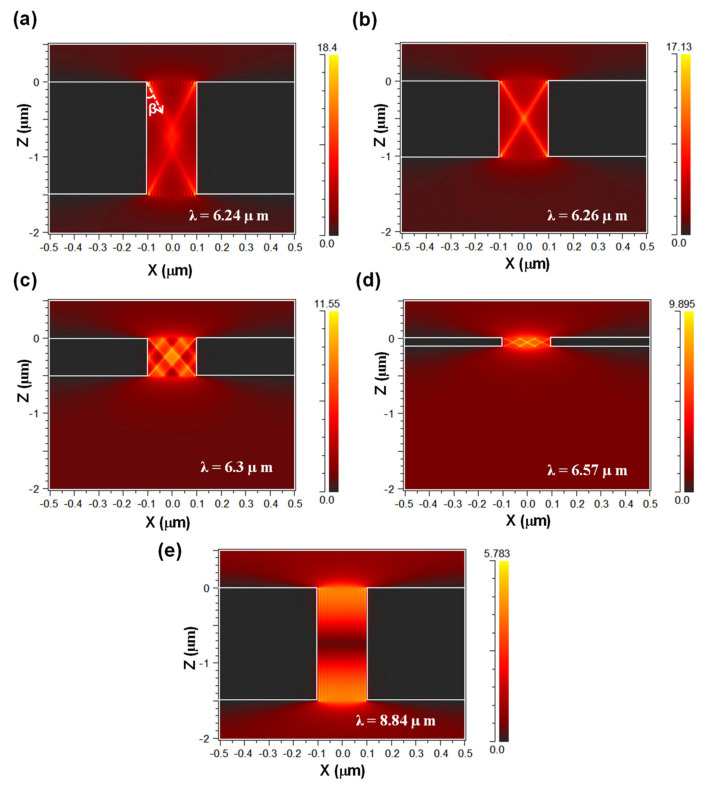
Amplitude of electric field distribution with *d* and the wavelength of (**a**) 1.5 μm and 6.24 μm, (**b**) 1.0 μm and 6.26 μm, (**c**) 0.5 μm and 6.3 μm, (**d**) 0.1 μm and 6.57 μm, and (**e**) 1.5 μm and 8.84 μm. The color scale represents the electric field amplitude.

The electric field (|E_x_|) distributions were calculated to identify these modes. The calculated |E_x_| distributions of the HPP modes are labeled as (i)–(iv) in Figure 5a, with the *d* values and wavelengths of each mode being (i) 1.5 μm and 6.24 μm, (ii) 1.0 μm and 6.26 μm, (iii) 0.5 μm and 6.3 μm, and (iv) 0.1 μm and 6.57 μm, respectively. For reference, the |E_x_| distribution of SPP2, denoted as mode (v) in Figure 5a, was calculated at a *d* of 1.5 μm and wavelength of 8.84 μm. Figure 6a–e show the calculated amplitude of E_x_ for modes (i)–(v) in Figure 5a. Please note that the scales of the x- and z-axes are not the same to clarify the resonance modes.

Figure 6a–d clearly show the unique property of HPPs in hBN, where the confined light in the slits travels in a zigzag trajectory. The propagation begins and ends at the two corners of the upper and lower nanoslits because the electric field is strongly concentrated on the corner of the nanoslits. Figure 6e shows that SPP2 is attributed to the conventional Fabry–Pérot resonance of SPRs [40].

As shown in Figure 6a–d, the zigzag trajectory is symmetric across the geometric center of the slit, indicating the formation of a Fabry–Pérot-like resonance. The propagation angle is defined as *β*, as shown in Figure 6a. The resonance order is defined as (*m*, *n*), where *m* and *n* are the resonance order coordinates in the x- and z-directions, respectively. The resonance order corresponds to the number of zigzags. The (*m*, *n*) triplets derived from Figure 6a–d for modes (i)–(iv) are (1, 1), (1, 1), (3, 2), and (3, 1), respectively. The resonance in hBN primarily depends on the aspect ratio of the hBN cavity rather than its size or shape [9,52]. Therefore, the aspect ratios of nanoslits, defined as *A* and *β* in [9,23], are given by

(2) $$\frac{1}{A}=\frac{w}{d}\sim \;\frac{m}{n}\tan\beta ,$$

(3) $$\tan\beta =\sqrt{-\frac{\epsilon_{⊥}}{\epsilon_{∥}}}.$$

$\epsilon_{∥}$ and $\epsilon_{⊥}$ were calculated from Equation (1), and tan *β* was calculated from Equation (3). *m/n* was then calculated from Equation (2). The obtained *m/n* are 0.9, 1.0, 1.5, and 3.2 for modes (i)–(iv), respectively. These values coincide well with the calculated results of 1, 1, 1.5, and 3 from Figure 6a–d, respectively. The differences between the analytically and numerically determined values for modes (i)–(iv) are slightly larger than those for the other two modes. The |E_x_| distribution shows that the zigzag pattern is slightly distorted at the center of the hBN cavity (Figure 6a) and slightly extends outside the hBN cavity (Figure 6d) owing to the extremely small cavity size. These differences are likely to be related. These results confirm that EOT occurs in the type-II RS band and is attributed to the Fabry–Pérot-like resonance induced by the hybridization of HPPs in hBN and SPRs in 1D nanoslits. Furthermore, it depends primarily on the aspect ratio of the hBN cavity, and not on its shape or size. The hybridization of HPPs in hBN and SPRs in 1D nanoslits can produce EOT with an extremely small cavity size because of the strong light confinement effect of hBN. As demonstrated in previous studies on hBN absorbers [22,23,24,25,26], hBN with plasmonic structures can enhance transmission with an extremely small hBN size.

## 4. Conclusions

The transmission of embedded hBN in 1D nanoslits was investigated. EOT was produced in the type-II RS band of hBN, as well as in other wavelength regions. In wavelength regions other than the type-II RS band, EOT is attributed to conventional Fabry–Pérot-like resonance of SPRs in 1D nanoslits, where the EOT wavelength is proportional to the slit depth. In contrast, in the type-II RS band, HPPs and SPRs strongly couple and produce Rabi splitting. The unique Fabry–Pérot-like resonance is induced by the hybridization of HPPs in hBN and SPRs in 1D nanoslits. The electric field distribution shows a zigzag trajectory, and the resonance number inside the hBN cavity is determined primarily by the aspect ratio of the 1D nanoslits and not by its size or shape. The slit size can be significantly reduced because of the strong light confinement effect of hBN. Such an EOT is also independent of the slit width and incident angle of light. The results obtained in this study can contribute to the development of improved strategies for the extreme confinement of IR light and can be applied in wavelength- or polarization-selective filters for advanced IR imaging [53] and other optical devices.

## Data Availability

No new data were created or analyzed in this study.
